# LLM-Assisted Scoping Review of Artificial Intelligence in Brazilian Public Health: Lessons from Transfer and Federated Learning for Resource-Constrained Settings

**DOI:** 10.3390/ijerph23010081

**Published:** 2026-01-07

**Authors:** Fabiano Tonaco Borges, Gabriela do Manco Machado, Maíra Araújo de Santana, Karla Amorim Sancho, Giovanny Vinícius Araújo de França, Wellington Pinheiro dos Santos, Carlos Eduardo Gomes Siqueira

**Affiliations:** 1 Department of Biomedical Engineering, Geoscience and Technology Center, Federal University of Pernambuco (UFPE), Recife 50740-550, PE, Brazil; maira.araujosantana@ufpe.br (M.A.d.S.); karla.amorims@ufpe.br (K.A.S.); wellington.santos@ufpe.br (W.P.d.S.); 2 Department of Science and Technology, Vice-Ministry of Science, Technology and Innovation, Brazilian Ministry of Health, Brasília 70719-040, DF, Brazil; giovanny.franca@saude.gov.br; 3 School of Dentistry, University of São Paulo (USP), São Paulo 05508-000, SP, Brazil; gabriela.manco.machado@usp.br; 4 School for the Environment, University of Massachusetts Boston (UMass Boston), Boston, MA 02125, USA; carlos.siqueira@umb.edu

**Keywords:** artificial intelligence, machine learning, transfer learning, federated learning, health systems

## Abstract

**Highlights:**

**Public health relevance—How does this work relate to a public health issue?**

- Maps how artificial intelligence is currently applied within the Brazilian Unified Health System (SUS), identifying structural gaps between diagnostic innovation and health system integration.
- Examines Transfer Learning and Federated Learning as practical responses to public health challenges such as data scarcity, privacy protection, and infrastructure limitations in the Global South.

**Public health significance—Why is this work of significance to public health?**

- Demonstrates that resource-aware AI architectures can enable equitable innovation in large universal health systems without reliance on centralized data extraction or high-cost infrastructure.
- Provides empirical evidence that data sovereignty and cooperative AI development are achievable within real-world public health settings through decentralized and adaptive methodologies.

**Public health implications—What are the key implications or messages for practitioners, policy makers and/or researchers in public health?**

- For policymakers, the findings support Federated Learning as a governance-aligned strategy consistent with data protection laws (e.g., LGPD) and national digital health sovereignty.
- For researchers and practitioners, the review highlights Transfer Learning and Federated Learning as scalable, low-resource pathways to deploy clinically relevant AI tools in underserved and heterogeneous health systems.

**Abstract:**

Artificial intelligence (AI) has become a strategic technology for global health, with increasing relevance amid the climate emergency and persistent digital inequalities. This study examines how AI has been applied in Brazilian healthcare through a scoping review with an in-depth methodological synthesis, focusing on Transfer Learning (TL) and Federated Learning (FL) as approaches to address data scarcity, privacy, and technological dependence. We searched PubMed, SciELO, and the CNPq Theses and Dissertations Repository for peer-reviewed studies on AI applications in Brazil, screened titles using AI-assisted tools with manual validation, and analyzed thematic patterns across methodological and infrastructural dimensions. Among 349 studies retrieved, six explicitly used TL or FL. These techniques were frequently implemented through multi-country research consortia, demonstrating scalability and feasibility for collaborative model training under privacy constraints. However, they remain marginal in mainstream practice despite their ability to deploy AI solutions with limited computational resources while preserving data sovereignty. The findings indicate an emerging yet uneven integration of resource-aware AI in Brazil, underscoring its potential to advance equitable innovation and digital autonomy in health systems of the Global South.

## 1. Introduction

### 1.1. Asymmetries of AI in Global Health

Over the past decade, artificial intelligence has advanced global healthcare by enhancing diagnostics, treatment, and health surveillance. AI-driven diagnostic platforms, such as robot-assisted microscopy for malaria detection, illustrate how low-cost automation can strengthen disease surveillance in regions facing shifting vector dynamics and endemic infectious burdens [1,2].

In critical care, researchers are developing translation tools powered by language models to bridge linguistic barriers during rapid-onset health crises, thereby improving coordination and data sharing in intensive care units (ICUs) [3]. At the environmental level, machine learning models are now predicting urban air quality with fine spatial granularity by coupling atmospheric physics with computational intelligence, enabling earlier interventions against environmental pollution-related cardiopulmonary risks [4,5]. Furthermore, equity-focused analyses of occupational safety emphasize how AI integration must be guided by justice to prevent exacerbating existing health disparities [6]. Validation studies show that machine learning outperforms traditional regression in detecting nonlinear links between environmental exposures and morbidity, making it a more flexible tool for data-driven policy decisions [7].

In high-income countries, large-scale multimodal biomedical AI models are showing promise in improving diagnoses, personalizing treatments, and streamlining clinical workflows across fields such as oncology, cardiology, and mental health [8]. These technologies often leverage vast datasets that include medical imaging, genomic profiles, electronic health records, and patient-reported outcomes [9]. However, the Transformer model architecture, self-supervised learning, and multimodal data inputs require massive Graphic Processing Unit (GPU) and computational power [9].

These advances are particularly relevant for high-burden chronic conditions, where early detection and tailored intervention can dramatically alter outcomes. Cardiovascular diseases (CVDs), for example, illustrate our argument; they remain the leading cause of mortality worldwide [10], and their burden is increasingly shaped by social and environmental determinants [11,12], such as air pollution, urbanization, and rising food insecurity [13]. In this evolving epidemiological landscape, artificial intelligence offers powerful tools for risk prediction, early diagnosis, and personalized management. Recent advances in large language models and multimodal AI allow integrated analysis of clinical, genomic, imaging, biosensor, and contextual data to accurately assess risk and guide interventions [14].

AI foundation models developed in leading academic and corporate research centers now integrate diverse data streams to provide real-time decision support and prognostic insight in high-income countries [15]. In contrast, their implementation in Global South exposes a landscape of both opportunity and vulnerability, where innovation meets the persistent constraints of infrastructure, equity, and governance. In the context of global health asymmetries, nations that dominate AI development will also possess the most advanced diagnostic and preventive capabilities [1]. López et al. [16] warn that technological dependency on foreign vendors risks reproducing existing global inequities. They advocate for a transition toward AI sovereignty grounded in the use of locally curated datasets and context-sensitive algorithms. This may strengthen national autonomy and resilience in digital health.

### 1.2. Unique Challenges in Brazil

The integration of artificial intelligence in Brazil is inextricably linked to the operational reality of the Brazilian Unified Health System (*Sistema Único de Saúde*, SUS), one of the world’s largest universal public health systems. Unlike the fragmented or insurance-based models often seen in high-income nations, SUS operates under the constitutional principles of universality, equity, and integrality, serving a continental population with varying degrees of digital maturity.

In 2024, Brazil committed BRL 2.3 billion over four years to boost its AI capabilities, funding a sovereign supercomputer and public sector projects in health, education, and social inclusion. At the core of this initiative lies the principle of “*IA para o bem de todos*” (AI for the common good), which positions technological progress to foster equity, autonomy, and sustainable development [17].

This vision is being operationalized in healthcare through the consolidation of the SUS Digital Program, which emerged from two decades of regulatory maturation in telehealth [18]. As Haddad et al. [18] explain, Brazil’s digital health transformation began with early teleconsultation programs under *Telessaúde Brasil Redes* (Brazil’s Telehealth Network). It advanced to a new phase in 2023 with the creation of the Secretariat of Information and Digital Health (*Secretaria de Informação e Saúde Digital*—SEIDIGI), a vice-ministerial body dedicated to information and digital health.

In 2024, the Ministry of Health launched the *Meu SUS Digital* (My Digital SUS) through ministerial ordinances. The program seeks to expand infrastructure, promote data interoperability, and ensure the ethical use of AI across all levels of the health system.

The integration of artificial intelligence into the Brazilian Unified Health System (SUS) faces structural hurdles that differ markedly from those in high-income nations, especially given the system’s continental scale and the heterogeneity of its digital maturity. Catapan et al. [19] show that while basic teleconsultations quickly grew during the COVID-19 pandemic, the shift to a comprehensive digital health ecosystem is still unfinished. This evolution is constrained by persistent regional asymmetries in infrastructure, connectivity, and interoperability within municipal and state administrations.

Emerging evidence from global surveys confirms these barriers. Cabral et al. [20] show that researchers worldwide identify interoperability limitations, data-quality issues, and ethical–regulatory uncertainties as the principal obstacles to safe AI integration in diagnostic medicine, reinforcing how the Brazilian case reflects global structural challenges.

The adoption of AI in Brazil raises critical concerns regarding data sovereignty and the commodification of public health, as emphasized by Modolo et al. [21]. Their analysis suggests that mHealth apps and digital health systems risk turning SUS into a large source of biological and behavioral data for private companies, leading to algorithmic automation of medical knowledge. This “platformization” risks subordinating clinical reasoning to opaque, foreign-owned algorithmic systems that neither reflect local epidemiological patterns nor incorporate the social determinants of health that shape illness in Brazil. Such dependence on black-box models threatens to reproduce and amplify inequities, including algorithmic biases affecting marginalized populations. Consequently, the challenge for Brazil extends far beyond technological acquisition: it requires building “sovereign AI” architectures capable of processing sensitive data locally, protecting citizens personal data, and preserving the ethical principles of universality, equity, and integrality that ground the SUS [21].

### 1.3. Transfer Learning and Federated Learning Solutions

Certain methodological advances in AI offer promise for reducing dependency on large datasets and high-cost infrastructure. Transfer Learning (TL) is a machine learning paradigm that allows models trained on one dataset or domain to transfer acquired knowledge to a different but related context. Instead of requiring models to be trained from scratch, TL leverages pre-trained representations to reduce computational burden, training time, and data dependency [22,23].

In healthcare, TL has proven effective for enhancing diagnostic and prognostic modeling in resource-constrained settings, allowing researchers in low- and middle-income settings to adapt models originally developed in high-income countries to their own clinical conditions [24,25]. For instance, de Araújo et al. [26] proposed a TL framework for early sepsis detection in intensive care units. Their model, trained on sparse ICU datasets, achieved high predictive accuracy Area Under the Curve (AUC) = 0.77) and enabled clinicians to anticipate systemic deterioration hours before standard clinical signs appeared, demonstrating the potential of TL to optimize critical care resources.

Meanwhile, FL has become a key strategy for protecting data privacy while enabling collaborative model development. Instead of pooling data in a single repository, institutions train models locally and share only the resulting parameters and updates. This process allows each site to contribute to the model’s collective improvement while keeping sensitive patient information securely within its own environment [27,28].

Federated Learning (FL) is an emerging machine learning paradigm specifically designed to enable collaborative model development under strict data protection constraints. In contrast to centralized methods, which consolidate sensitive datasets within a single repository, federated learning enables institutions to conduct model training locally using their own data. Only model parameters or updates are shared with a coordinating server, enhancing privacy and security. This decentralized training process enables collective learning while preserving patient confidentiality, institutional autonomy, and compliance with data protection regulations, making FL particularly relevant for healthcare applications [27,28].

Federated Learning adopts a decentralized training architecture that enables health systems in resource-constrained environments in the Global South to overcome structural limitations related to data fragmentation, privacy, and infrastructure. In such contexts, clinical data are often fragmented across multiple institutions, interoperability remains limited, and legal or ethical considerations may restrict the sharing of data. Decentralized architectures reduce the need for large-scale data transfers, lower infrastructure requirements, and allow institutions to retain full control over their datasets. Recent methodological advances, such as Equitable Federated Learning with Neural Cellular Automata (FedNCA), further adapt FL to resource-constrained settings by addressing data heterogeneity, limited connectivity, and imbalances in data quantity across sites [29].

Brazil’s participation in international federated learning initiatives illustrates the practical feasibility of this approach in real-world public health research. Studies in cardiovascular disease and cancer imaging have shown that FL can achieve robust predictive performance while keeping sensitive clinical data within local institutions [28,30]. These experiences indicate that FL architectures can be integrated into large public health systems like the Brazilian SUS, allowing local data to advance global AI development while preserving data sovereignty [1].

For instance, global genomic research is shifting toward sending code to data instead of centralizing large datasets, as data silos and privacy rules make aggregation challenging [31]. This structural imperative for decentralization parallels a broader distortion in medical AI: the prevailing narrative frequently exhibits a “complexity bias,” privileging massive Deep Learning architectures while overlooking computationally efficient alternatives that are often better suited for resource-constrained environments like the Brazilian Unified Health System. This asymmetry is clearly demonstrated by the limited adoption of “Shallow AI” paradigms, such as Single Layer Extreme Learning Machines (ELM). These models have shown notable efficiency in essential applications—including rapid COVID-19 diagnosis through hybrid architectures, real-time epileptic seizure detection on edge devices, and precise tumor classification [32]. Nevertheless, they remain less utilized compared to resource-intensive foundation models that require substantial GPU resources. This preference for algorithmic depth over utility mirrors the challenges faced by the Global South, where the uncritical adoption of high-resource models threatens to deepen technological dependence. Consequently, achieving digital sovereignty requires a pivot toward methodologies that prioritize adaptation and privacy over raw computational power.

Brazil’s digital health transformation provides the context for this study. We use a scoping review to map how AI is currently applied in the national health system. We then evaluate the specific contributions of TL and Federated FL. Our mapping shows that these techniques are still emerging in Brazil’s research output. Even so, they represent a strategic frontier for resource-constrained settings. By examining how these adaptive architectures function within Brazil’s complex and universal health system, this review offers evidence on how the Global South can move from technological dependence toward methodological and data sovereignty.

## 2. Methods

### 2.1. Search Strategy

We designed this study as a scoping review with a two-stage analytical design, combining (i) a thematic landscape mapping of AI applications in Brazilian healthcare and (ii) an in-depth methodological synthesis of studies using Transfer Learning (TL) or Federated Learning (FL), reported in accordance with PRISMA-ScR [33,34,35]. This approach comprises two distinct phases: (1) a Thematic Landscape Analysis (Scoping Phase) to map the broad distribution of AI applications in Brazilian healthcare, and (2) a In-depth methodological synthesis (In-Depth Sub-review Phase) of the six studies employing TL or FL. This dual structure allows for a quantitative overview of the national research ecosystem while providing a qualitative, mechanism-based evaluation of techniques capable of addressing resource constraints. A protocol was not prospectively registered; however, we report methods transparently and provide supporting materials, including the PRISMA-ScR checklist [36], in the Supplementary File S3. In addition, this scoping review was retrospectively registered on the Open Science Framework (OSF; https://doi.org/10.17605/OSF.IO/UYV2R, accessed on 21 October 2025).

We searched for studies on AI in Brazilian healthcare using three sources: PubMed, SciELO, and the CNPq Theses and Dissertations Repository. Each search string combined controlled vocabulary and free-text terms, including “artificial intelligence,” “health,” and “Brazil.” We adapted each query to the syntax of its respective database. Appendix A provides the complete search strategies. We completed the search in July 2025, retrieving a total of 1135 records. Following a data quality assessment, we identified and removed 15 duplicate records, resulting in a final screening pool of 1120 unique records (see Supplementary File S1). This pool served as the source for both the landscape map and the targeted sub-review.

To avoid inadvertently discarding relevant studies due to inconsistent metadata across heterogeneous sources (e.g., institutional repositories, databases with overlapping records, or variations in titles and authorship), we retained all entries without automatic deduplication. This conservative approach aimed to maximize sensitivity during the initial screening phase, especially given the variability in reporting standards across Brazilian research outputs. Because many CNPq records lacked abstracts, we screened them solely by title. We exported all titles into a structured spreadsheet that included metadata such as year and source.

### 2.2. AI-Assisted Screening Workflow

We used the GPT-4o-mini language model via the OpenAI API to streamline the screening process and assess feasibility in low-resource environments.

The instruction prompt provided to the model was:

“You are a researcher participating in a systematic review. Your job is to screen studies and decide upon the inclusion and exclusion criteria below, if they should be included in the review. Make your decision only based on the given criteria, title and abstract. Answer it with ‘Include’ if the study should be included, and ‘Exclude’ otherwise.

Eligibility criteria: You want to map Artificial Intelligence projects, studies, and research done in Brazil.”

For the first phase (Thematic Landscape), we employed the GPT-4o-mini language model via the OpenAI API to streamline the initial relevance screening. A zero-shot classification prompt in Portuguese instructed the model to label each title based on a single inclusion criterion: ‘Does this study discuss artificial intelligence applied to healthcare in Brazil?’ This broad criterion ensured the capture of the full landscape of applications, independent of the specific machine learning technique used.

We submitted titles in batches of 10 using Google Colab on a standard CPU laptop, without GPU acceleration or fine-tuning. Appendix B describes the full implementation. To maximize sensitivity, we treated all “Maybe” responses as “Include.” This conservative approach ensured that we did not overlook borderline cases during the AI-assisted screening phase.

The AI-assisted screening functioned as a semantic rather than merely lexical filter, distinguishing between general digital health initiatives and applied computational intelligence. The model excluded studies on digitization, like telemedicine logistics or electronic health records, unless they included predictive modeling or algorithmic analysis. It applied a domain-specific filter that differentiated between agricultural biology and One Health applications, retaining environmental studies only when they demonstrated clear public health relevance. Beyond simple exclusion, the model enriched the dataset by generating the thematic classifications used in our landscape analysis, effectively acting as a preliminary technical reviewer that prioritized the intersection of algorithmic complexity and health outcomes.

#### 2.2.1. Human Validation and Consensus

After the model classified the titles, human reviewers independently validated records without access to the model’s decisions. Following this step, coauthors Borges FT and Sancho KA conducted comprehensive human oversight during three months (August to October 2025) of the screening process. The research team independently reviewed all records classified as relevant by the language model (n = 349) and discussed each case until reaching consensus regarding inclusion in the thematic landscape analysis.

The screening proceeded iteratively. During human review of records generated across screening reruns, the reviewers manually identified and removed 15 duplicate entries. In parallel, the reviewers examined records not selected by the AI and identified no additional unique studies meeting the inclusion criteria beyond those selected by the model. Accordingly, no article entered or exited the review process solely on the basis of an automated decision.

Because the reviewers applied consensus-based verification to all AI-included records, combined with human oversight of AI-excluded records and manual deduplication across reruns, the study did not calculate formal inter-rater reliability statistics (e.g., Cohen’s kappa). This approach minimized the risk of false negatives while maintaining transparency and methodological rigor consistent with scoping review practices.

#### 2.2.2. Researcher Expertise and Positional Transparency

Coauthors Borges FT and Sancho KA conducted the human validation and consensus review. Both authors are trained in public health and social sciences. Borges FT has worked in the assessment and deployment of artificial intelligence solutions in the Brazilian Unified Health System (SUS) since 2019, while Sancho KA has engaged in applied AI research since early 2023, with a focus on linguistic and epidemiological applications.

Both authors hold research fellowships from the Brazilian National Council for Scientific and Technological Development (CNPq) under the Technological Development Fellowship (*Bolsa de Desenvolvimento Tecnológico*) program and are affiliated with the Biomedical Computing Laboratory at the Federal University of Pernambuco (UFPE). Borges FT works on the deployment and evaluation of AI solutions within SUS, and Sancho KA, a linguistics specialist with a PhD in Epidemiology, focuses on the development of language-based systems and chatbots to support healthcare professionals in SUS.

The authors include this positional disclosure to ensure transparency regarding the epistemological background and applied expertise informing the human oversight and validation stages of the AI-assisted screening process.

### 2.3. Study Selection and Thematic Classification

Phase 1: Thematic Landscape Selection: Following the AI-assisted screening and human validation, we identified 349 studies (Supplementary File S2) meeting the general inclusion criteria (AI in Brazilian Health). These records were retained to map the thematic distribution of the field (results presented in Table 1), classifying studies into domains such as Diagnostic AI, Public Policy, and Health Research.

Phase 2: Rather than restricting the initial search to specific methodological terms (e.g., “Transfer Learning” or “Federated Learning”), we adopted a broad, high-sensitivity search strategy using high-level descriptors such as “Artificial Intelligence” and “Machine Learning.” This approach is consistent with the exploratory purpose of scoping reviews and accounts for the variability and incompleteness of metadata in studies from the Global South, where methodological details are often reported only in the full text.

To ensure that no TL or FL studies were missed within the 349 records included in the scoping set, we conducted an explicit verification pass across the entire dataset. This verification combined targeted keyword searches (e.g., “transfer learning,” “fine-tuning,” “pre-trained,” “federated,” “FedAvg,” “aggregation”) with manual review of titles, abstracts, and full texts where necessary. This process confirmed that only six studies explicitly implemented TL or FL as core methodological components with sufficient implementation detail to support comparative synthesis.

For the in-depth methodological synthesis, we applied the following criteria: (1) explicit use of TL or FL as a central methodological component, and (2) sufficient reporting of implementation details. This analytic focus selection identified six studies. The remaining 343 studies, while relevant to the Brazilian AI landscape, primarily relied on conventional architectures (e.g., standard convolutional neural networks, random forests) and were therefore not selected for the in-depth synthesis, but were retained in the scoping map.

We catalogued all 349 included studies in a standardized spreadsheet to support thematic mapping and transparency. The full list of studies is provided in Supplementary File S2, and their thematic distribution across application domains is summarized in Table 1. The six TL/FL studies were subsequently reviewed in full and analyzed as representative cases illustrating resource-aware, privacy-preserving AI strategies within the Brazilian public health context. Table 1 highlights studies implementing Transfer Learning or Federated Learning as a methodological attribute that cuts across clinical domains, rather than presenting them as a separate clinical category.

### 2.4. Role of AI and Risk Mitigation

The AI-assisted screening process was designed to support, not replace, human scientific judgment. The language model functioned exclusively as a decision-support tool to enhance efficiency during the preliminary title-screening phase. All inclusion and exclusion decisions remained under the responsibility of the human reviewers, who independently validated model outputs and resolved discrepancies by consensus. No study was included or excluded solely on the basis of an automated decision.

We put several safeguards in place to address possible risks from using AI-assisted screening, like model bias, mistakenly excluding candidates, or depending too much on standard terms. First, a deliberately broad inclusion criterion was applied at the AI-screening stage, and all borderline or uncertain classifications (“Maybe”) were conservatively retained for human review. Second, human reviewers evaluated a subset of titles independently and blinded to the model’s outputs to assess concordance and correct misclassifications. Third, the screening was limited to titles (and abstracts when available), avoiding downstream automation of full-text assessment or methodological appraisal.

Together, these measures ensured that AI assistance enhanced scalability and consistency while preserving human oversight, methodological integrity, and accountability throughout the review process.

## 3. Results

**Figure 1:**
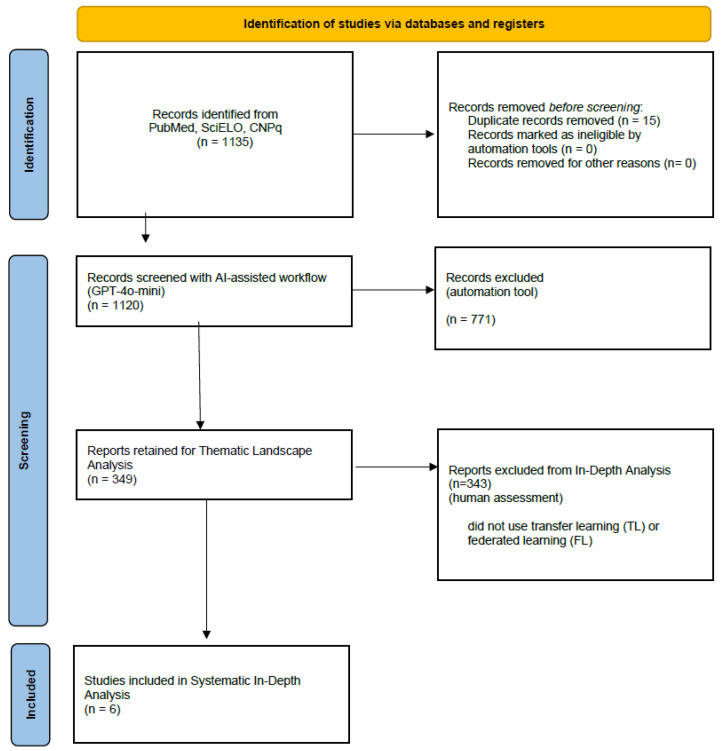
PRISMA-ScR flow diagram illustrating identification, screening, eligibility assessment, and analytic focus selection for the thematic landscape map and the in-depth methodological synthesis (n = 6) [35].

To visualize the selection process across both phases, we present a flow diagram shown in Figure 1. We began with a broad and inclusive search strategy to capture the full spectrum of artificial intelligence applications in Brazilian healthcare. Our goal was to understand the overall landscape before identifying specific methodological patterns. We did not set TL or FL as inclusion criteria; rather, they emerged from the analysis as central approaches addressing Brazil’s resource and infrastructure constraints. This exploratory design allowed us to compare national adoption patterns with those observed in high-income countries and to identify areas of convergence and persistent gaps in computational feasibility and scalability. By subsequently focusing on TL and FL, we were able to highlight AI innovations that respond to local healthcare needs rather than merely replicate global trends.

The thematic distribution of the 349 included studies is detailed in Table 1. The analysis reveals that the Brazilian AI landscape is predominantly centered on Pattern Recognition, which accounts for 89.1% of the identified literature. When divided into clinical subcategories, most studies align with national epidemiological priorities, focusing primarily on Infectious Diseases (such as COVID-19, Dengue, and Zika), followed by Oncology and Cardiovascular monitoring. This concentration suggests that AI research in Brazil has largely followed a “disease-specific” trajectory, leveraging deep learning primarily for diagnostic precision in high-burden conditions.

In contrast, research addressing the structural integration of AI into the public health system remains marginal. Only 6.9% of studies were classified under Applications in SUS, focusing on critical areas such as primary care triage, bed management, and family health logistics. This asymmetry indicates a gap between the maturity of diagnostic algorithms and their operational deployment within the Brazilian SUS. Within this context, studies utilizing Transfer Learning and Federated Learning, identified as a cross-cutting methodological attribute, represent a nascent but strategic methodological niche (1.7%), specifically targeting the barriers of data scarcity and infrastructure that the mainstream literature often overlooks.

The thematic and methodological mapping of the 349 included studies confirms that AI research in Brazilian healthcare is predominantly centered on pattern recognition, which accounts for 89.1% of the identified literature. This dominant landscape, or ‘mainstream’ research activity, relies heavily on standard deep learning architectures and traditional machine learning committees. In contrast, the application of TL and FL represents a marginal segment (approximately 2%) of the total output. This quantitative gap supports our choice to analyze these six studies, which, though statistically minor, address resource limits, data scarcity, and privacy concerns in the Global South, factors often missed in wider pattern recognition research.

Table 2 synthesizes the six studies selected for in-depth analysis, which explicitly implemented TL, FL, or related model adaptation strategies suited to data-scarce and infrastructure-constrained healthcare settings. These studies span diverse geographic and institutional contexts, with Brazilian participation ranging from local public health initiatives to global AI consortia. The table integrates methodological descriptions, dataset characteristics, performance indicators (such as AUC and Dice Similarity Coefficient), and public health implications, offering a consolidated view of how TL and FL techniques have been operationalized. For example, TL was leveraged to improve diagnostic automation in malaria microscopy and hypertension prediction in children, while FL enabled secure, cross-institutional modeling for cancer imaging and COVID-19 prognosis. Despite their relatively small share in the overall dataset (2%), these studies achieved strong technical performance and demonstrated real-world applicability—especially in domains requiring interoperability, privacy preservation, and adaptation to local epidemiological patterns. This synthesis highlights the strategic potential of TL and FL as context-sensitive AI architectures aligned with the operational realities of Brazil’s Unified Health System (SUS) and other Global South health systems.

We analyzed six academic studies on TL and FL. Six of these studies draw their data from South America, specifically from Dayan et al. [27], Lorenzer et al. [30], Sheller et al. [28], and Araújo Moura et al. [39]. The sixth study integrates U.S. data presented by Stanford et al. [38] and includes research contributions from a Brazilian academic institution within a consortium. This selection offers a balanced view across different regions while highlighting collaborative approaches in investigating TL and FL. As shown in Table 2, these initiatives range from single-institution experiments in TL [37,38] to complex, multi-institutional and FL consortia involving partners across three continents [28].

The study by Dayan et al. [27] implemented a FL framework across 20 international hospitals to predict clinical outcomes for COVID-19 patients, including in-hospital mortality. By training decentralized models across diverse institutions without transferring patient data, the authors demonstrated that FL achieved performance comparable to or superior to that of centralized models, with AUC scores exceeding 0.80 across multiple sites. This approach preserved data privacy while enabling cross-institutional learning, a significant advantage in contexts with fragmented health systems and privacy constraints, such as Brazil’s SUS.

However, the authors also noted challenges, including data heterogeneity, inconsistent clinical coding, and coordination overhead. Those barriers that would need to be addressed for successful implementation in resource-limited environments.

The study by Lorenzer et al. [30] focused on the foundational challenges of applying FL across international hospital networks by harmonizing electronic medical record (EMR) data from Austria, Germany, and Brazil. Their objective was to prepare a unified dataset for training models to predict major adverse cardiovascular events (MACE). Brazil’s contribution came from the Ribeirão Preto Medical School at the University of São Paulo (USP). Despite differences in coding systems and data granularity, the researchers successfully standardized features using international terminologies, such as the International Classification of Diseases, 10th Revision (ICD-10), Logical Observation Identifiers Names and Codes (LOINC), and Anatomical Therapeutic Chemical Classification System (ATC). Nevertheless, this process was labor-intensive and revealed deep structural challenges, especially in mapping medication and laboratory data, which required extensive manual work and clinical expertise. Their work provides a valuable blueprint for how institutions in Brazil and elsewhere might participate in federated modeling without compromising data privacy, but it also underscores the need for national-level infrastructure and semantic standardization for broader scalability.

In one of the earliest large-scale FL implementations in medical imaging, Sheller et al. [28] trained a tumor boundary segmentation model for glioblastoma using data from 17 institutions across North and South America, including the USP. The study aimed to address the challenge of rare cancer detection, where data scarcity is a persistent barrier to effective model training. By employing a federated architecture, the authors enabled collaborative learning without centralized data aggregation, achieving a mean Dice Similarity Coefficient (DSC) of 0.78, which was comparable to fully centralized training. The study also highlighted several limitations, including communication overhead, variability in data quality, and the complexity of model versioning. Despite that, the findings underscore the feasibility of privacy-preserving big data analytics for rare diseases, which often lack sufficient local data in middle-income countries like Brazil.

The study led by Ramos et al. [37] explores the use of TL to automate malaria diagnosis by detecting Plasmodium vivax in microscopic blood smear images using a dataset of 6222 Regions of Interest (ROIs). They primarily sourced from the Broad Bioimage Benchmark Collection (BBBC) and supplemented by locally collected images from the Brazilian Amazon. Then trained and evaluated six deep neural networks. Among them, DenseNet201 consistently outperformed others, achieving an AUC of 99.41% and demonstrating statistically superior performance across most classification metrics. Notably, the study incorporated rough circular segmentation and did not rely on manual feature engineering or complex preprocessing, enabling an efficient pipeline suitable for low-resource environments. By validating results through 100-fold cross-validation and including leukocyte images, a common source of diagnostic error, the study highlighted both robustness and generalizability. This work exemplifies how TL, when carefully tailored to local epidemiological and infrastructural conditions, can yield high-accuracy diagnostic tools with minimal computational cost.

Sanford et al. [38] investigated how to improve the generalizability of automated prostate segmentation models using TL and advanced data augmentation techniques. The study trained a deep learning model on 648 prostate MRI exams from a single center and tested it on five external datasets. A hybrid 2D-3D convolutional neural network (CNN), incorporating pre-trained ResNet-50 layers, was used for segmentation. The authors applied “deep stacked transformation” (DST) as a domain-specific data augmentation strategy and also fine-tuned the model using samples from the target institutions. These combined approaches improved the Dice Similarity Coefficient (DSC) by up to 2–3% for both whole-prostate and transition-zone segmentations, with final mean DSCs reaching 91.5 and 89.7, respectively. Notably, the model demonstrated resilience across diverse MRI vendors, scanning protocols, and institutional settings, highlighting TL as a key enabler of robust AI tools in heterogeneous, and resource-diverse healthcare systems.

Araujo-Moura et al. [39] applied TL to enhance hypertension prediction among South American children and adolescents using multicenter data from the SAYCARE study. Initially, models trained on a children’s dataset achieved high accuracy (>0.90), but showed poor generalizability to the adolescent sample due to limited and more variable data. By transferring knowledge from the well-performing child model to adolescent predictions using CatBoost, Random Forest, and other tree-based algorithms, researchers significantly improved model performance, achieving an AUC-ROC of 0.82 for CatBoost. The study further employed SHAP analysis to interpret the contribution of individual variables, revealing that lifestyle factors such as soft drinks, chips, and filled cookies consumption were strong predictors of elevated blood pressure. This work highlights how TL not only improves model accuracy in data-scarce settings but also helps reveal context-sensitive risk factors that may support public health interventions across multiple countries, including in Global South.

## 4. Discussion

### 4.1. Limitations and Strengths

We recognize several limitations in this study. We restricted our analysis to peer-reviewed academic publications, which may exclude relevant gray literature and ongoing projects within government or industry. We analyzed only studies published in English and Portuguese, which may have led to the omission of research disseminated in other languages. Although our narrative synthesis allowed us to identify key methodological and contextual trends, it did not include a quantitative meta-analysis, which limits the ability to measure effect sizes or statistical associations. Our interpretation also depends on the quality of reporting within the included studies. Incomplete metadata or inconsistent terminology may have affected comparability across sources.

As a scoping review, this study relies on qualitative thematic synthesis rather than statistical meta-analysis. Accordingly, the identification of TL and FL as cross-cutting methodological attributes reflects an exploratory mapping of approaches within the Brazilian AI landscape, rather than a definitive causal or quantitative assessment.

We used AI-assisted title screening to increase efficiency and consistency during the initial selection phase. However, automated classifiers can prioritize familiar terminology and overlook interdisciplinary or locally grounded studies that use less standardized language. The algorithm may also inherit linguistic and geographic biases from its training data, overrepresenting research from high-income settings and underrepresenting studies from Global South contexts. Although we manually validated to correct these issues, we acknowledge that AI-based screening remains sensitive to data composition and parameter settings, which can affect inclusiveness and reproducibility.

We designed this study for full reproducibility by conducting all analyses on standard CPU-based computing environments. This approach shows that researchers can generate meaningful bibliometric and methodological insights without relying on high-performance hardware. By using accessible infrastructure, we reproduced the real computational conditions of many research institutions in low- and middle-income countries and demonstrated that rigorous AI research remains feasible under resource constraints.

A key strength of this study lies in its broad and inclusive search strategy, which captured the full spectrum of artificial intelligence applications in Brazilian healthcare before narrowing the focus to transfer and federated learning. This approach allowed emergent identification rather than predefined selection of relevant methodologies, strengthening the validity and comprehensiveness of the analysis. By situating TL and FL within the wider national landscape, the study provides a more contextual and representative understanding of Brazil’s AI adoption patterns, avoiding the bias of topic-restricted reviews.

### 4.2. Comparative Challenges: Data-Centric vs. Infrastructure-Centric Barriers

While both TL and FL offer viable pathways to reduce technological dependence in resource-constrained settings, our review reveals that they face distinct implementation barriers. The challenges associated with TL are primarily data-centric, revolving around the quality of representation and domain adaptation. In contrast, the challenges of FL are infrastructure-centric, driven by the complexities of orchestration, connectivity, and semantic interoperability.

Table 3 shows that TL methods by Ramos et al. [37] and Sanford et al. [38] mainly face issues with “domain shift,” where differences in image staining, protocols, or definitions between source and target domains reduce performance. The primary mitigation strategy here is the curation of annotated local datasets for fine-tuning, which, while computationally lighter than full training, still requires significant manual effort in labeling.

Conversely, FL initiatives like the FeTS and PRE-CARE ML consortia face “systemic heterogeneity”. The hurdles here are not just statistical but logistical: communication latency, software versioning across disparate IT systems, and the rigorous demand for semantic harmonization of Electronic Medical Records (EMR) before any training can occur. Lorenzer et al. [30] demonstrated that the most resource-intensive aspect of FL is not the algorithmic computation but the mapping of local clinical terminologies (e.g., heterogeneous ICD-10 or drug codes) to a unified global ontology.

The six studies share a goal: creating AI models that adapt to context instead of copying approaches from high-resource environments. All highlight challenges including scarce data, varied conditions, and limited computational resources in applying AI to health. Together, they represent a new approach to context-aware innovation, using adaptable methods to address longstanding technological inequities in the Global South.

The experience of the reviewed consortia confirms that interoperability is not merely a technical step but a governance challenge. While the literature often frames interoperability as a matter of software compatibility, the practical struggles reported by Lorenzer et al. [30] within the PRE-CARE ML project reveal that the true barrier is semantic divergence. Federated Learning works best when a “semantic governance” framework (such as pre-established data ontologies like LOINC and SNOMED-CT) ensures all decentralized nodes are mathematically and clinically compatible before deployment.

Furthermore, the issue of privacy must be reframed from a purely cryptographic problem to one of national sovereignty. Modolo et al. [21] warn that without robust governance, the digitalization of health systems risks turning the SUS into a source of biological and behavioral data for private companies, leading to the commodification of public health data. In this context, Federated Learning emerges as a structural solution to this political risk. By design, FL architectures like those used in the EXAM and FeTS consortia align with the sovereignty principles advocated by the Brazilian General Data Protection Law (LGPD). They allow Brazilian institutions to participate in global scientific advances without relinquishing custody of their datasets. Thus, privacy in the Global South context is not just about concealing patient identity but about retaining control over the value and usage of population health data against extractive practices.

### 4.3. Regulatory Implications: LGPD and ANVISA

The Brazilian General Personal Data Protection Law (LGPD), Law 13.709/2018 [40], along with guidelines from the Brazilian Health Regulatory Agency (ANVISA), creates a unique structure for AI implementation that supports decentralized architectures. The General Personal Da imposes strict requirements on the processing of sensitive personal data regarding health. Federated Learning aligns naturally with the LGPD’s principle of data minimization and purpose limitation, as it avoids the creation of massive centralized data lakes that are vulnerable to breaches. By keeping patient data within the originating institution (e.g., a specific hospital in the SUS network), FL reduces the legal friction associated with Data Transfer Impact Assessments (DPIA).

This decentralization introduces novel paradigms for the validation frameworks overseen by the Brazilian Health Regulatory Agency (ANVISA). While current RDC regulations for Software as a Medical Device (SaMD) provide a robust structure for the validation of fixed datasets, FL and TL introduce dynamic models where the training data (in FL) remains local, or the model (in TL) evolves through fine-tuning. To fully leverage these technologies, there is an opportunity to expand the regulatory perspective toward ‘lifecycle regulation.’ This approach would focus on validating the training process and monitoring local performance continuously, serving as a complement to the established pre-market validation of static global models.

## 5. Conclusions

Artificial intelligence has become a highly sensitive technology for public health, particularly in the context of the climate emergency. Dependence on high-income countries that control AI deployment risks deepens existing inequities in access and governance. Yet, our review shows that a Global South country like Brazil has begun to adapt pre-trained models developed elsewhere and integrate them into their health systems. Both transfer learning and federated learning are emerging through transnational and interinstitutional collaborations, demonstrating scalability and feasibility in low-resource contexts. The limited number of studies employing these techniques should not discourage their use but instead signal the need for sustained investment in local capacity, ethical governance, and cooperative research infrastructures. Building such frameworks can transform technological dependence into shared sovereignty, ensuring that AI development advances equity, resilience, and public health security in an era of global interdependence.

Future research should focus on developing resource-aware, and scalable AI strategies that perform reliably under data scarcity, computational limitations, and infrastructural diversity typical of Global South health systems. Few existing studies address these challenges directly, and even fewer provide validated frameworks for integrating such models into public health governance. There is also a need for systematic evaluation of FL and TL in real-world clinical workflows, particularly within primary care and population surveillance programs. Future research can examine how cooperative governance models, which integrate national autonomy with international collaboration, may affect data sovereignty and innovation across health systems of varying capacities.

## Figures and Tables

**Table 1:** In-depth methodological synthesis of studies explicitly implementing Transfer Learning or Federated Learning in Brazilian health research.

Thematic Domain	Subcategory/Application Area	Count	TL Studies	FL Studies	Technique Highlight
Pattern Recognition	Infectious Diseases (COVID-19, Zika, Dengue, Malaria)	110	1	1	Deep Learning (CNNs), Transfer Learning (Microscopy), Federated Learning (Prognosis)
	Oncology (Breast, Prostate, Skin, Lung cancer)	86	1	1	CNNs, Segmentation (U-Net), Federated Learning (Rare Cancers), Transfer Learning
	Cardiovascular (ECG, Heart Disease risk)	46	1	1	ML Classifiers, Signal Processing, Transfer Learning (Pediatrics), Federated Learning (Harmonization)
	Neurological (Alzheimer’s, Stroke, Parkinson’s)	38	0	0	MRI analysis, Deep Learning
	General Medical Imaging (X-ray, CT, Ultrasound)	36	0	0	Image Processing, Computer Vision
Applications in SUS	Primary Care & Family Health	15	0	0	Risk Stratification, Triage Algorithms (incl. Low-resource ML)
	Hospital Management & Logistics	10	0	0	Resource Allocation, Bed Management
Public Policies	Regulation & Ethics	6	0	0	Qualitative Analysis, Framework Proposals
Health Research	Methodological Studies	3	0	0	Reviews, Benchmarking
Challenges	Implementation Barriers	2	0	0	Surveys, Critical Analysis
TOTAL		352	3	3	

Author’s note: The total count of thematic classifications (352) slightly exceeds the number of unique studies (349) due to three studies receiving multiple thematic tags. Percentages are calculated based on the unique study count (n = 349). The “Studies employing TL/FL” theme exclusively contains the studies employing FL and TL identified for in-depth analysis. Source: Developed by the authors based on the scoping review.

**Table 2:** Summary of included studies employing Transfer Learning and Federated Learning: Scope, methodology, and public health impact.

Study and Brazilian Participation	Context and Dataset Characteristics	Methodology (AI Technique)	Performance and Quantitative Indicators	Clinical and Public Health Relevance
Dayan et al. [27] (Global Consortium). Brazil Role: DASA & hospitals among 20 global sites.	Scope: 20 institutions across 4 continents (N. America, S. America, Europe, Asia). Data: Electronic Medical Records (EMR) + Chest X-rays (CXR).	FL: EXAM model training decentralized models without data transfer.	AUC: >0.92 (avg across sites). Gen: +38% improvement in generalizability over local models.	Resource Allocation: Predicts oxygen requirements (24–72 h), optimizing ICU/ventilator usage during pandemic surges.
Lorenzer et al. [30] (ERA PerMed Consortium). Brazil Role: Ribeirão Preto Medical School (USP).	Scope: Multicenter data from Austria, Germany, and Brazil. Data: Unharmonized EMRs predicting MACE.	Federated Learning (Interoperability): Semantic harmonization of disparate coding systems (ICD-10, LOINC, ATC).	Outcome: Successful semantic mapping across 3 languages/systems; data ready for federated modeling.	Interoperability Blueprint: Establishes standards for cross-border public health surveillance without sharing sensitive patient data.
Sheller et al. [28] (FeTS Initiative). Brazil Role: University of São Paulo (USP).	Scope: 71 institutions across 6 continents. Data: 6314 patients (largest dataset for Glioblastoma).	Federated Learning: U-Net architecture with local normalization for tumor boundary detection.	DSC: 0.78 (mean). Gain: +33% boundary delineation vs. public models; +20% perf. over local models.	Rare Disease Precision: Enables big data analytics for rare cancers where single-center data is insufficient for training.
Ramos et al. [37] (Single-Center Initiative). Brazil Role: Fiocruz Rondônia (Amazon Region).	Scope: Local samples from Brazilian Amazon + Broad Bioimage Benchmark. Data: 6222 Regions of Interest (ROIs).	Transfer Learning: DenseNet201, InceptionV3, MobileNetV2 adapted for malaria diagnosis.	AUC: 99.41%.Accuracy: 97.29% (DenseNet201).	Diagnostic Automation: Automates diagnosis in low-resource settings, reducing dependency on specialists in endemic regions.
Sanford et al. [38] (NIH/NVIDIA Collaboration). Brazil Role: Hospital Albert Einstein (São Paulo).	Scope: 6 international centers (USA, Brazil, UK, Italy). Data: 648 MRI exams used to test generalizability.	Transfer Learning: AH-Net with deep augmentation and fine-tuning for prostate segmentation.	DSC: 91.5 (Whole Prostate); 89.7 (Transition Zone). Gain: +2–3% DSC via TL.	Oncology Workflow: Enhances generalizability of models across different hospitals/scanners for radiotherapy planning.
Araujo-Moura et al. [39] (SAYCARE Study). Brazil Role: USP & UEPB coordination.	Scope: Multicentric study across 7 South American cities. Data: 658 participants (351 children, 307 adolescents).	Transfer Learning: Deep neural networks and fine-tuning for pediatric hypertension prediction.	Accuracy: 1.0 (Adolescents with TL); ~0.90 (Children). Gain: +12% F1-score with TL.	Early Prevention: Identifies risk factors (e.g., processed food) early, enabling targeted interventions in schools/families.

Author’s note: Abbreviations: AUC: Area Under the Curve; DSC: Dice Similarity Coefficient; EMR: Electronic Medical Records; FL: Federated Learning; MACE: Major Adverse Cardiovascular Events; TL: Transfer Learning. Source: Developed by the authors based on the scoping review.

**Table 3:** Comparative analysis of implementation challenges between Transfer Learning and Federated Learning in resource-constrained settings.

Dimension	Transfer Learning	Federated Learning
Primary Barrier	Domain Shift & Negative Transfer: Performance degradation when source data (e.g., HIC datasets) differs statistically from target data (e.g., Amazonian samples).	Client Heterogeneity (Non-IID): Divergence in data distribution and quantity across participating institutions destabilizes model convergence.
Infrastructure Needs	Low to Moderate: Can often be deployed on standard hardware after pre-training; computation is localized and offline.	High: Requires reliable high-speed connectivity to handle communication overhead, latency, and synchronization between global nodes.
Data Interoperability	Label Alignment: Requires target labels to match source classes (e.g., exact tumor types or parasite species).	Semantic Alignment: Requires strict harmonization of ontologies (ICD-10, LOINC, ATC) across all nodes to ensure the algorithm “reads” data identically.
Privacy Risk	Centralization Risk: Often requires moving local data to a central server or cloud for fine-tuning, potentially exposing patient information.	Inference Risk: Data remains local, but the model is susceptible to gradient leakage or reconstruction attacks if differential privacy is not strictly enforced.
Governance Focus	Asset Management: Focuses on acquiring and licensing pre-trained models and datasets.	Orchestration: Focuses on managing the consortium, legal agreements for weight sharing, and software versioning across borders.

Source: Developed by the authors based on the narrative thematic synthesis.

## Data Availability

All data supporting the findings of this study are available within the article and its Supplementary Materials. The datasets generated and analyzed during the study—including the structured bibliographic database of 1135 retrieved records and the curated list of 349 studies on artificial intelligence in Brazilian healthcare—are provided as Supplementary Files S1 and S2. All primary records were retrieved from publicly accessible databases (PubMed, SciELO, and the CNPq Theses and Dissertations Repository). No new individual-level or confidential data were created or analyzed.

## Supplementary Materials

The following supporting information can be downloaded at: https://www.mdpi.com/article/10.3390/ijerph23010081/s1. Supplementary File S1: Spreadsheet of AI in Health studies in Brazil (comprehensive dataset of 1135 records screened). Supplementary File S2: Relevant studies on AI in Health in Brazil (detailed list of the 349 included studies with thematic and methodological classifications). Supplementary File S3: Preferred Reporting Items for Systematic reviews and Meta-Analyses extension for Scoping Reviews (PRISMA-ScR) Checklist.

## Abbreviations

The following abbreviations are used in this manuscript:

AI	Artificial Intelligence
ANVISA	Agência Nacional de Vigilância Sanitária
AUC	Area Under the Curve
ATC	Anatomical Therapeutic Chemical Classification System
BBBC	Broad Bioimage Benchmark Collection
CPU	Central Processing Unit
CVD	Cardiovascular Disease
DST	Deep Stacked Transformation
DPIA	Data Transfer Impact Assessments
DSC	Dice Similarity Coefficient
EMR	Electronic Medical Record
FedNCA	Equitable Federated Learning with Neural Cellular Automata
FeTS	Federated Tumor Segmentation
FL	Federated Learning
GPU	Graphics Processing Unit
HE	Homomorphic Encryption
HIC	High-Income Country
ICD	International Classification of Diseases
LLM	Large Language Model
LOINC	Logical Observation Identifiers Names and Codes
MACE	Major Adverse Cardiovascular Event
MCTI	Ministry of Science, Technology and Innovation (Brazil)
ML	Machine Learning
PNS	*Pesquisa Nacional de Saúde* (National Health Survey, Brazil)
PRE-CARE ML	Predictive Care through Machine Learning (ERA PerMed Consortium)
RF	Random Forest
SaMD	Software as a Medical Device
SEIDIGI	Secretaria de Informação e Saúde Digital
SHAP	SHapley Additive exPlanations
SMPC	Secure Multi-Party Computation
SUS	*Sistema Único de Saúde* (Brazilian Unified Health System)
TL	Transfer Learning
XGBoost	Extreme Gradient Boosting

## Appendix A

### Search Strategy

**Table A1:** Search strategy.

Database (Platform)	String Used
PubMed (MEDLINE)	(“artificial intelligence”) AND (“health” OR “healthcare” OR “public health” OR “medicine” OR “medical”) AND Brazil)
SciELO	(“inteligência artificial”OR “machine learning”) AND (“saúde” OR “saúde pública” OR “health” OR “healthcare”) AND (Brasil OR Brazil)
CNPq Theses & Dissertations Repository	(“inteligência artificial”OR “machine learning”) AND (“saúde” OR “saúde pública” OR “health” OR “healthcare”) AND (Brasil OR Brazil)

Source: Developed by the authors based on narrative thematic synthesis.

## Appendix B

### Prompt and Technical Setup for AI-Assisted Screening

To enhance transparency and reproducibility, this appendix provides the full prompt, technical environment, and implementation details used for the AI-assisted screening process.

Prompt used for zero-shot classification:

text

Classify the article titles below as “Include”, “Maybe”, or “Exclude” for a systematic review on the use of artificial intelligence in healthcare in Brazil.

For each title, respond in the format:

Number: <title number>

Classification: <Include|Maybe|Exclude>

Justification: <brief explanation>

Titles:

[Numbered list of titles]

Respond in the same order as the titles.

The prompt was delivered in Portuguese to match the language of the majority of retrieved titles and maintain the model’s comprehension accuracy. It was submitted in batches of 10 titles per request, with the expectation that the model would respond using a structured format.

Technical implementation:

- Model: GPT-4o-mini
- Access method: OpenAI API
- Environment: Google Colab (https://colab.research.google.com/)
- Hardware: Standard laptop using CPU (no GPU acceleration)
- Programming language: Python 3.10+
- Key libraries: openai, pandas, re, math, time
- Batch size: 10 titles per request
- Output: CSV file with model classification and justification for each title

The Python script used to automate the process included:

- Dynamic prompt construction for each batch
- Submission of prompts via OpenAI API
- Parsing of structured responses using regular expressions
- Integration of results into a working dataset for analysis

A two-second delay between batches was added to reduce the likelihood of API rate limits.

As described in the Methods section, all titles labeled as “Maybe” were conservatively treated as “Include” for comparison with human reviewers and to calculate inter-rater agreement metrics.

The full code is available upon request for academic and research purposes.
